# Diffusion Tensor Imaging Studies on Spontaneous Subarachnoid Hemorrhage-Related Brain Injury: A Mini-Review

**DOI:** 10.3389/fneur.2020.00283

**Published:** 2020-04-28

**Authors:** Min Kyeong Cho, Sung Ho Jang

**Affiliations:** Department of Physical Medicine and Rehabilitation, College of Medicine, Yeungnam University, Daegu, South Korea

**Keywords:** subarachnoid hemorrhage, diffusion tensor imaging, diffusion tensor tractography, brain injury, neural injury

## Abstract

Accurate diagnosis of the presence and severity of neural injury in patients with subarachnoid hemorrhage (SAH) is important in neurorehabilitation because it is essential for establishing appropriate therapeutic strategies and developing a prognosis. Diffusion tensor imaging has a unique advantage in the identification of microstructural white matter abnormalities which are not usually detectable on conventional brain magnetic resonance imaging. In this mini-review article, 12 diffusion tensor imaging studies on SAH-related brain injury were reviewed. These studies have demonstrated SAH-related brain injuries in various neural tracts or structures including the cingulum, fornix, hippocampus, dorsolateral prefrontal region, corticospinal tract, mamillothalamic tract, corticoreticular pathway, ascending reticular activating system, Papez circuit, optic radiation, and subcortical white matter. We believe that these reviewed studies provide information that would be helpful in science-based neurorehabilitation of patients with SAH. Furthermore, the results of these reviewed studies would also be useful for clarification of the pathophysiological mechanisms associated with SAH-related brain injury. However, considering the large number of neural tracts or neural structures in the brain, more research on SAH-related brain injury in other neural tracts or structures should be encouraged.

## Introduction

Spontaneous subarachnoid hemorrhage (SAH), which involves the extravasation of blood into the subarachnoid space between the arachnoid membrane and the pia mater covering the brain, mainly occurs following rupture of an aneurysm, and comprises 5% of all cases of stroke (1, 2). Various neurologic complications are known to occur in more than 50% of patients with SAH, including cognitive impairment, motor weakness, consciousness impairment, visual problem, and somatosensory deficit (3–16). Accurate diagnosis of the presence and severity of neural injury in patients with SAH is important in neurorehabilitation because it is essential for establishing therapeutic strategies and predicting a patient's prognosis. Previous studies have demonstrated the presence of SAH-related brain injury by using functional magnetic resonance imaging (MRI), positron emission tomography, or single-photon emission computed tomography (17–20). However, these imaging methods are limited when localizing a specific neural tract or structure in the brain.

Diffusion tensor imaging (DTI) has a unique advantage in the identification of microstructural white matter abnormalities that are not usually detectable on conventional brain MRI (21–23). DTI allows evaluation of the integrity of the brain's white matter to be determined by virtue of its ability to image water diffusion characteristics (24–28). Among the various DTI parameters that can be examined, fractional anisotropy (FA), mean diffusivity (MD, or apparent diffusion coefficient [ADC]), and tract volume (TV, or fiber volume or fiber number) parameters are the most commonly used (24–27). The FA value indicates the degree of directionality of water diffusion such as that associated with the axon, myelin, and microtubule. Therefore, a reduced FA value indicates injury to white matter microstructures (24–27). The MD value indicates the magnitude of the water diffusion and an increment of MD suggests vasogenic or cytotoxic edema or the presence of cellular debris accumulation following a neural injury (24–27). The TV (or fiber volume or fiber number) value reflects the number of voxels (values on a regular grid within a three-dimensional space) included in a neural tract, thereby indicating the total number of neural fibers within the tract (24–27). A low TV value suggests a low number of neural fibers within a neural tract, which indicates an injury of that neural tract (28–30). Diffusion tensor tractography (DTT), which is a derivative of diffusion tensor imaging (DTI), enables the three-dimensional visualization and estimation of neural tracts (122,831). The advantage of DTT over DTI is that the characteristics of an entire neural tract can be determined by examining DTT parameters and analyzing the reconstructed configuration of the tract (31).

To date, the pathophysiological mechanisms of SAH-related brain injury have not been clearly clarified. However, several mechanisms have been suggested: global vasogenic edema in both white and deep gray matter, vasospasm and cerebral ischemia, mechanical injury (*via* increased intracranial pressure or direct mass effect by SAH), and chemical injury (a blood clot can cause neural injury by release of potentially damaging substances, such as free iron, which may result in the generation of free radicals or inflammatory cytokines) (3, 5, 32–37).

**Figure 1:**
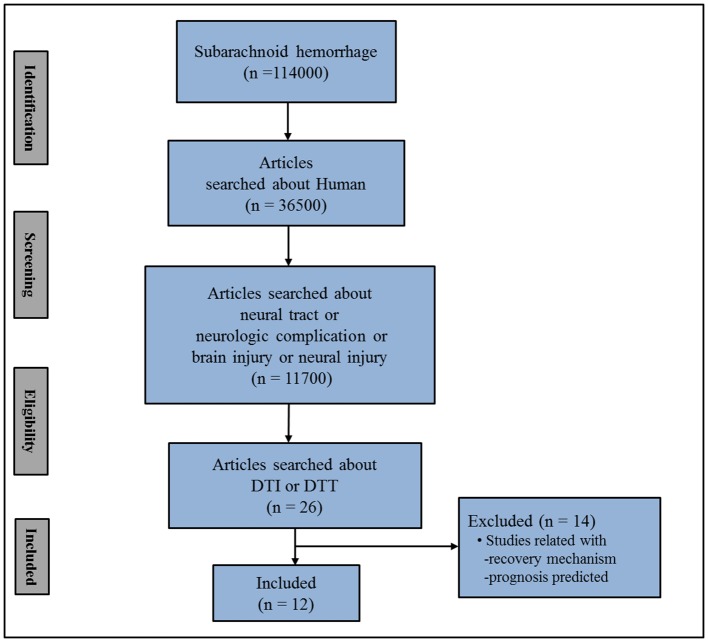
Flow diagram for the selection of studies for review. SAH; subarachnoid hemorrhage.

In this mini-review, DTI studies on spontaneous SAH-related brain injury are reviewed. Relevant studies in the period from 1990 to 2019 were identified by accessing the following electronic databases: PubMed, Google Scholar, and Web of Science. Additionally, the following keywords/abbreviations were used when searching the databases: DTI, DTT, SAH, neural tract, neural injury, neurologic complication, and brain injury. This review was limited to studies of human subjects with spontaneous SAH. In addition, we excluded studies that did not demonstrate a definite brain injury due to the lack of normal control data (38–44). We selected relevant studies based on the flow diagram shown in Figure 1. Overall, 12 studies were selected and reviewed (3–14) (Table 1).

**Table 1:** Diffusion tensor imaging-based studies of spontaneous subarachnoid hemorrhage-related brain injury.

**Authors**	**Number of patients**	**Duration to initial DTI**	**Pathology & lesion location**	**Analyzed neural structure**	**DTT methods**	**Limitations**
Hong et al. (3)	11	54.1 days (29-97)	ACoA (11 patient)	Cingulum, Fornix	Configuration DTT parameters (FA↓, MD↑, TV↓)	No detailed neuropsychological tests, Small number of subjects
Schweizer et al. (4)	1	303 days	Not described	Hippocampus, dorsolateral Prefrontal lesion	Configuration DTT parameters (FA↓)	No control data, case study
Yeo et al. (5)	22	7.3 ± 3.1 weeks	Not described	Corticospinal tract	DTT parameters (FA↓: midbrain) MD(-)	No analysis of other motor tracts
Jang et al. (6)	16	5.7 ± 1.5 weeks	Not described	Mammillothalamic tract	DTT parameters (FA↓,TV↓)	No detailed neuropsychological tests
Jang et al. (7)	17	6.2 weeks (3-10)	ACoA:14pts PCoA:1pt ACA:1pt ICA:1pt	Corticoreticular pathway	DTT parameters (FA↓, MD↑) FV↓	No detailed analysis of other motor tracts
Jang and Kim (8)	24	6.49 weeks (3.14–13.86)	ACoA:14pts PCoA:3pts MCA:2pts Other:5pts	Lower ARAS	DTT parameters (FA:-, MD:-) TV↓	No analysis of other ARAS
Jang and Yeo (9)	1	3 months	ACoA	Papez circuit	Configuration DTT parameters (FA↓, MD↑, FV↓)	Case study
Jang and Seo (10)	1	4 weeks	PCoA	Optic radiation	(FA-,ADC-, FN↓)	Case study
Jang et al. (11)	21	5.9 weeks (3-12)	ACoA:15pts ACA:3pts PCoA:1pt other	Optic radiation	DTT parameters (FA↓, ADC↑,FN:-)	No visual field test
Darwazeh et al. (12)	21	8.92 ± 2.4 weeks	Not described	Mammillothalamic tract	DTT parameters (FA↓, ADC↑)	No detailed neuropsychological tests
Jang and Yeo (13)	1	9 weeks	MCA bifurcation	Precommissural Fornix	Configuration DTT parameters (FA↓, MD↑, FV↓)	Case study
Reijmer et al. (14)	49:SAH 22:unruptured aneurysm	2 weeks 6 months	Not described	White matter	DTT parameters (FA-, MD↓)	Non-specific DTI analysis

*DTI, diffusion tensor imaging; DTT, diffusion tensor tractography; ACoA, anterior communicating artery; FA, fractional anisotropy; MD, mean diffusivity; TV, tract volume; PCoA, posterior communicating artery; ACA, anterior cerebral artery; ICA, Internal carotid artery; FV, fiber volume; MCA, middle cerebral artery; ARAS, ascending reticular activating system; ADC, apparent diffusion coefficient; FN, fiber number; SAH, subarachnoid hemorrhage*.

## Studies of Spontaneous Subarachnoid Hemorrhage-Related Brain Injury Demonstrated Through Diffusion Tensor Imaging

In 2012, Hong et al. demonstrated injuries of the cingulum and fornix in patients with SAH due to rupture of an anterior communicating artery (ACoA) aneurysm (3, 45). Eleven patients were scanned at an average of 54.1 days after onset, and six (54.5%) and seven (63.6%) of the 11 patients exhibited discontinuations at the anterior cingulum and the fornical body, respectively, on DTT. The FA and TV values for the cingulum and fornix were comparatively low, whereas the MD value was relatively high, except for the MD value of the left fornix. Although the patients showed cognitive impairment on the Mini-Mental State Examination (MMSE; average, 23.3; full score, 30; cutoff score, <25, higher score means better cognition), cognitive impairment was not shown to be correlated with any of the DTT parameters (46). The authors suggested that the cingulum and fornix in patients with SAH due to an ACoA aneurysm rupture appeared to be injured via mechanical means related to the hematoma or to the concurrent cerebral infarct. The authors concluded that the anterior cingulum and fornix can be vulnerable to injury by SAH following the rupture of an ACoA aneurysm; on that basis, they recommended using DTT to evaluate the cingulum and fornix in patients with an ACoA aneurysm rupture (3). However, their patients were not evaluated using detailed neuropsychological tests and the study included a relatively small number of subjects, thus limiting the application of the results.

During the same year, Schweizer et al. (4) reported on a patient with long-term memory impairment, as measured by a delayed recall on the California Verbal Learning Test, and impaired planning abilities, as measured by the Stockings of Cambridge Test, subsequent to a perimesencephalic SAH (4). Conventional brain MRI performed 303 days after onset did not reveal an abnormality, and the patient's hippocampal volumes on conventional brain MRI were in the above-average range on both sides. By contrast, DTT revealed that, compared to the left hippocampus, the right hippocampus had a notably lower FA value and a smaller bundle of white matter tracts. In addition, the left dorsolateral prefrontal region had a lower FA value than that of the same region on the right side. The authors concluded that the patient's long-term memory impairment may be related to the reduced hippocampal white matter integrity, whereas the patient's impaired planning may be related to the reduced white matter integrity in the left dorsolateral prefrontal region (4). However, this study had limitations in that it was a case report without normal control data. Furthermore, the authors did not reconstruct the neural tracts precisely.

**Figure 2:**
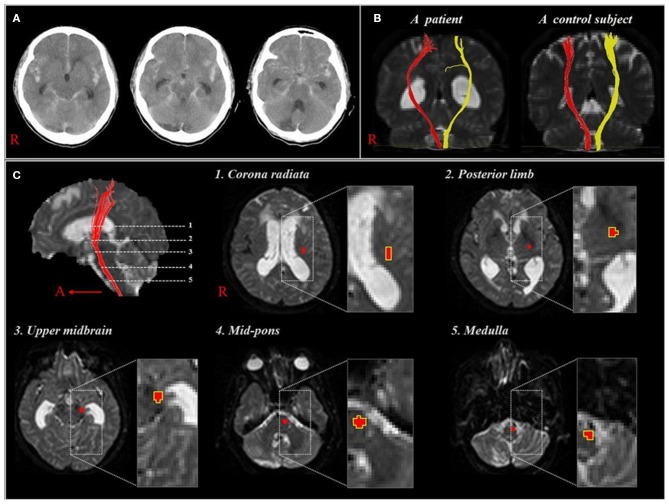
**(A)**, CT showing a subarachnoid hemorrhage of one patient at onset (58 year-old woman). **(B)**, Diffusion tensor tractographies for the corticospinal tract of the patient shown in **(A)** and one normal control subject (41 year-old man). **(C)**, Images show 5 regions of interest along the corticospinal tract pathway [reprinted with permission from Yeo et al. (5)].

Subsequently, but during the same year, Yeo et al. reported on corticospinal tract (CST) injuries in patients with SAH (5). Twenty-two patients with mild motor weakness (Motricity Index: average, 74.2; full score, 100, higher score means better motor function) without definite lesions along the CST pathway or on the fronto-parietal cortices related to motor execution (47). The DTI data were acquired at an average of 7.3 weeks after onset and the FA and ADC values were measured at five regions of interest along the CST pathway from the corona radiata to upper medulla (Figure 2). The FA value for the CST area of the midbrain was lower in the patient group compared with the control group, with no further significant changes in other regions. Consequently, the authors suggested that the CST could be vulnerable to SAH in the midbrain area because SAH frequently occurs in a perimesencephalic cistern, and the location of the CST is close to this cistern at the midbrain (35, 48). In addition, the authors assumed that the CST injury in the midbrain area could occur through mechanical injury due to increased intracranial pressure or a direct mass or through chemical injury due to the free iron in the blood clot (34). The authors concluded that the CST injury in the midbrain area appears to be one of the pathophysiological mechanisms resulting in motor weakness in SAH (5). However, this study was limited because the authors did not analyze other neural tracts that are involved in motor function, such as the corticoreticular pathway and rubrospinal tract.

In 2014, Jang et al. demonstrated injury of the mammillothalamic tract (MTT), a portion of the Papez circuit that connects the mammillary body and the anterior thalamus, and they reported that MTT injury severity was correlated with cognitive impairment in patients with SAH (6). Sixteen patients with SAH underwent DTI at an average of 5.7 weeks after onset. The FA and TV values for the MTTs in the patient group were lower than those in the control group. In addition, MMSE scores for the patients revealed a strong positive correlation with TV. Individual analyses revealed that 10 (62.5%) of the 16 patients showed MTT abnormalities related to DTT parameters and/or configuration. The authors assumed that the MTT in patients with SAH may be injured by mechanisms similar to those of a CST injury in the midbrain region because the MTT is closely located to the perimesencephalic cistern (5, 35). However, this study was limited because the cognitive impairment was not evaluated using detailed neuropsychological tests.

In 2015, Jang et al. reported on corticoreticular pathway injuries in 17 patients with motor weakness but confirmed intact integrity of the CST following SAH (7). DTI scanning was performed at an average of 6.2 weeks after onset. The configurational analysis revealed that 12 (70.6%) of 17 patients and 18 (52.9%) of 34 hemispheres exhibited discontinuations of the corticoreticular pathway in the midbrain area. The motor function of the contralateral shoulder, hip, and total lower extremity of the discontinued corticoreticular pathway, which was measured by determining the Motricity Index, were lower than those of the intact corticoreticular pathway. In addition, the corticoreticular pathway in the patient group had lower FA and fiber volume values and a higher MD value. Moreover, the FA value of the corticoreticular pathway showed a moderate positive correlation with the Motricity Index of the proximal joint muscles (shoulder and hip). As a result, the authors concluded that corticoreticular pathway injury may be common in patients with motor weakness of the proximal joint muscles after SAH, and the injury appeared to be related to weakness in the contralateral shoulder, hip, and lower extremity. The authors assumed that the corticoreticular pathway could be damaged mechanically or chemically by hemorrhage in the subarachnoid area of the midbrain although that pathway is located more deeply than the CST in the midbrain. As a result, the authors recommended that patients with SAH who show unexplained proximal weakness or leg weakness should have the corticoreticular pathway evaluated using DTI (7). However, this study was limited because the authors did not analyze the other neural tracts that are involved in motor function, such as the corticoreticulospinal and rubrospinal tracts.

In 2015, Jang and Kim demonstrated injury of the ascending reticular activating system (ARAS) in the brainstems of 24 patients with SAH due to aneurysmal rupture (8). DTI data were acquired at an average of 6.49 weeks after onset and were used to reconstruct the ARAS between the reticular formation of the pons and the intralaminar nuclei of the thalamus. The TV of the ARAS was lower in the patient group, and the Glasgow Coma Scale scores of the patients had a strong positive correlation with the TV of the ARAS (49). The authors assumed that ARAS injury might be the result of the hematoma in the subarachnoid space around the thalamus and brain stem. As a result, the authors recommended that DTT should be used to analyze the ARAS in patients with impaired consciousness after SAH (8). However, the authors did not analyze the other neural tracts that are involved in consciousness, thereby limiting the study.

In 2016, Jang and Yeo reported on a Papez circuit injury in a patient with provoked confabulation—a memory disturbance occurring in clear consciousness—in association with disruption of retrieval and encoded memory quality after SAH (9, 50). Since SAH onset, the patient presented severe memory impairment and confabulation was provoked through questioning. The patient showed severe memory impairment on Memory Assessment Scale results: short-term memory (65: 1%), verbal memory (58: <1%), visual memory (60: <1%), and global memory (58: <1%) (51). Furthermore, the patient exhibited provoked confabulation upon provocation testing (52). On 3 month DTT, the DTT parameters and the reconstructed tract configuration revealed injuries in each neural tract of the Papez circuit (thalamocortical tract, fornix, MTT, and cingulum). The authors suggested that extensive and multiple neural injuries of the Papez circuit in this patient might be related to the provoked confabulation as well as to the severe memory impairment (39). However, because it is a case report, generalization from this study is limited.

In 2017, Jang and Yeo reported on a patient who presented with a visual field defect and optic radiation injury following SAH due to the rupture of an aneurysm in the left posterior communicating artery (10). The patient presented with a visual field defect, although there was no medical problem identified in either eye. Peripheral field defects were assessed in both eyes using the Humphrey visual field test at 4 weeks after onset. On 4 month DTT, fiber number reductions in both optic radiations of the patient were detected. On DTT reconstruction of the optic radiations of the patient, both optic radiations were thinner than those in normal subjects. As a result, the authors recommended that DTT-based evaluation of the optic radiations is necessary for patients who complain of a visual field defect following SAH (10). However, this study is limited to further generalization because it is a single case study.

In 2018, Jang et al. used DTI to investigate the optic radiation in patients with SAH following the rupture of a cerebral artery aneurysm. The authors recruited 21 patients with aneurismal SAH who showed no definite lesions along the visual pathway on the conventional brain MRI. The DTI data were acquired at an average of 5.9 weeks after onset (11). The decreased FA value and increased ADC value without significant difference in the fiber number were observed in the optic radiation of the patients (11). The authors had assumed that SAH may cause injury to the OR, which is located near the arachnoid space in the occipital lobe. However, presence of a visual defect due to OR injuries was not confirmed by the clinical evaluations performed in this study. Regardless, because visual field defect is one of the various clinical manifestations observed in patients with SAH, the authors recommended a thorough evaluation of possible optic radiation injury in patients with SAH.

In 2018, Darwazeh et al. demonstrated that the MTT was more vulnerable than the CST to the effects of SAH in 21 patients with good outcomes (Grade 5 on Glasgow Outcome Scale at 3 months) (12). The DTI scanning was performed at an average of 8.92 weeks after onset in the 21 SAH patients. The decrement of FA value and increment of ADC value of the MTT without significant difference in the CST indicates the MTT injury were observed on DTT. In addition, the FA value of the MTT was positively correlated with the MMSE score. Therefore, the authors concluded that patients with good outcomes after SAH appeared to suffer an injury of the MTT without an associated injury of the CST. The authors assumed that the MTT is more affected by SAH than the CST because the MTT is thinner than the CST and because some portion of the MTT is more exposed than the CST to SAH in the perimesencephalic cistern (12). However, this study had limitations because the cognitive impairment was not evaluated using detailed neuropsychological tests. In addition, although the authors assessed the CST, this study only recruited patients with very mild motor weakness, which is not usually associated with the CST injury (average Motricity Index: 95.07, full score: 100).

In 2018, Jang and Yeo reported on a patient who showed memory impairment and injury of the precommissural fornix after SAH due to the rupture of a right middle cerebral artery bifurcation aneurysm (13). Memory impairment at 9 weeks after onset was characterized as follows: MMSE: 24, and Memory Assessment Scale: short-term memory (score of 70, 2%), verbal memory (score of 82, 12%), visual memory (score of 79, 8%), and global memory (score of 77, 6%) (51). On a 9-week multifiber DTT assessment, the postcommissural fornix showed discontinuations and the FA and fiber volume values for the precommissural fornix were lower, but without significance, that those of the postcommissural fornix. Consequently, the authors concluded that this patient's memory impairment (severe short-term memory impairment) appeared to be related to injury to the precommissural fornix, which supplies cholinergic innervation from the cholinergic nuclei (the medial septal nucleus and vertical nucleus of the diagonal band) in the septal region, whereas the postcommissural fornix is mainly involved in the transfer of information related to episodic memory (53, 54). The authors recommended that separate DTT evaluations of the precommissural and postcommissural fornices would be useful in patients with memory impairment following SAH (13). However, this study is limited because it is a single case study.

In 2018, Reijmer et al. investigated differences in white matter microstructure between patients with SAH and patients with an unruptured intracranial aneurysm (14). Forty-nine patients with aneurysmal SAH and 22 patients with an unruptured intracranial aneurysm underwent DTI twice (at 2 weeks and 6 months after onset). Cognition was evaluated 3 months after onset (11 subtests covering five cognitive domains [language, memory, executive functioning, attention, and visuospatial function] using the Boston Naming Test, the Rey Auditory Verbal Learning Task-Dutch version immediate and delayed recall test, the Rey-Osterrieth Complex Figure Test for copy and delayed recall conditions, the Category Fluency Test, the Letter Fluency Test, the Digit Span Test of the Wechsler Adult Intelligence Scale III or IV, the Go/No-Go task of the Frontal Assessment Battery test, the Visual Elevator Task, and the Symbol Substitution task of the Wechsler Adult Intelligence Scale III or IV. The patients with SAH had higher MD in the white matter which indicated they had more white matter microstructural abnormality than patients with an unruptured aneurysm at 2 weeks after onset. However, the MD value io the SAH patients returned to the level of the unruptured intracranial aneurysm group at 6 months after onset, and there was no significant difference in the FA values obtained from the two DTI scans. The MD value abnormalities at 2 weeks after onset in the patients with SAH were associated with impaired performance on the Boston Naming Test (language) and the Rey Auditory Verbal Learning Task-Dutch version immediate recall test (immediate memory). As a result, the authors concluded that, in the subacute post-onset phase, patients with SAH have temporary white matter abnormalities that are associated with the cognitive impairments in the chronic stage of SAH (14). However, this study was limited because the authors did not analyze specific brain areas that were relevant to their clinical evaluation.

## Conclusions

In this mini-review article, 12 DTI-based studies on SAH-related brain injury were reviewed (3–14). The studies focused on SAH-related brain injury in various neural tracts or structures including the cingulum, fornix, hippocampus, dorsolateral prefrontal region, CST, MTT, corticoreticular pathway, ARAS, Papez circuit, optic radiation, and white matter (3–14). We believe that these reviewed studies are helpful in developing a scientific basis for neurorehabilitation in patients with SAH because a precise diagnosis of the presence and severity of a neural injury is essential for successful neurorehabilitation. In addition, the results of these reviewed studies are useful for further elucidation of the pathophysiological mechanisms associated with SAH-related brain injury. However, considering the number of neural tracts and neural structures in the brain, much more research on SAH-related brain injury, particularly of other neural tracts or structures, should be encouraged. Furthermore, four of the 12 reviewed studies were case reports; therefore, further studies involving large numbers of subjects are warranted.

## Author Contributions

MC: study concept, design, and critical revision of manuscript for intellectual content. SJ: study concept and design, manuscript development, writing, funding, and critical revision of manuscript for intellectual content.

## Conflict of Interest

The authors declare that the research was conducted in the absence of any commercial or financial relationships that could be construed as a potential conflict of interest.
